# Per- and polyfluoroalkyl substances mixture exposure is associated with colorectal cancer TNM stage

**DOI:** 10.3389/ftox.2026.1904192

**Published:** 2026-08-11

**Authors:** Ning Kang, Yang Zhao, Zhi Huang, Rongbin Li, Xiaoqian Ma, Shigang Ding, Fei Li, Shengju Yin, Weiwei Fu

**Affiliations:** 1 Department of Maternal and Child Health, School of Public Health, Shanghai Jiao Tong University School of Medicine, Shanghai, China; 2 Ministry of Education-Shanghai Key Laboratory of Children’s Environmental Health, Xinhua Hospital, Shanghai Jiao Tong University School of Medicine, Shanghai, China; 3 Department of Laboratory Medicine, Peking University Third Hospital, Beijing, China; 4 Department of Gastroenterology, Peking University Third Hospital, Beijing, China; 5 Beijing Key Laboratory for Helicobacter Pylori Infection and Upper Gastrointestinal Diseases, Beijing, China; 6 Ministry of Education-Shanghai Key Laboratory of Children’s Environmental Health and Department of Developmental and Behavioural Paediatric and Child Primary Care, Xinhua Hospital Affiliated to Shanghai Jiao Tong University School of Medicine, Shanghai, China

**Keywords:** colorectal cancer, HFPO-DA, mixture exposure, per- and polyfluoroalkyl substances, TNM stage

## Abstract

Environmental pollutants have been implicated in colorectal cancer risk, but the association between plasma per- and polyfluoroalkyl substance (PFAS) concentrations and TNM stage remains unclear. We recruited 110 patients with colorectal cancer in Beijing, China, between March and June 2024. Plasma concentrations of 32 PFAS were measured, and 23 PFAS with detection rates greater than 70% were included in the analysis. Random forest analysis was used to identify the highest-ranking PFAS for TNM-stage classification, followed by multivariable ordinal logistic regression and restricted cubic spline models to evaluate single-PFAS associations and concentration–response patterns. Bayesian kernel machine regression was further applied to assess the joint associations of the selected PFAS mixture, and stratified analyses were conducted by sex and age. PFOA and n-PFOS showed the highest median concentrations, at 2.899 ng/mL and 2.444 ng/mL, respectively. Random forest analysis identified PFPeS, PFTrDA, PFTeDA, HFPO-DA and 8:2 Cl-PFESA as five highest-ranking PFAS for TNM-stage classification. In single-pollutant models, HFPO-DA was positively associated with more advanced TNM stage (odds ratio 2.76, 95% confidence interval 1.39–5.46; P = 0.004), with a linear concentration–response association suggested by restricted cubic spline analysis (overall P = 0.012). Mixture analysis further indicated that higher combined PFAS concentrations were associated with more advanced TNM stage, with a more pronounced positive mixture–response pattern among female patients. These findings suggest that higher plasma PFAS concentrations, particularly HFPO-DA, may be associated with more advanced TNM stage in colorectal cancer and warrant confirmation in larger prospective studies.

## Graphical Abstract

**Figure:**
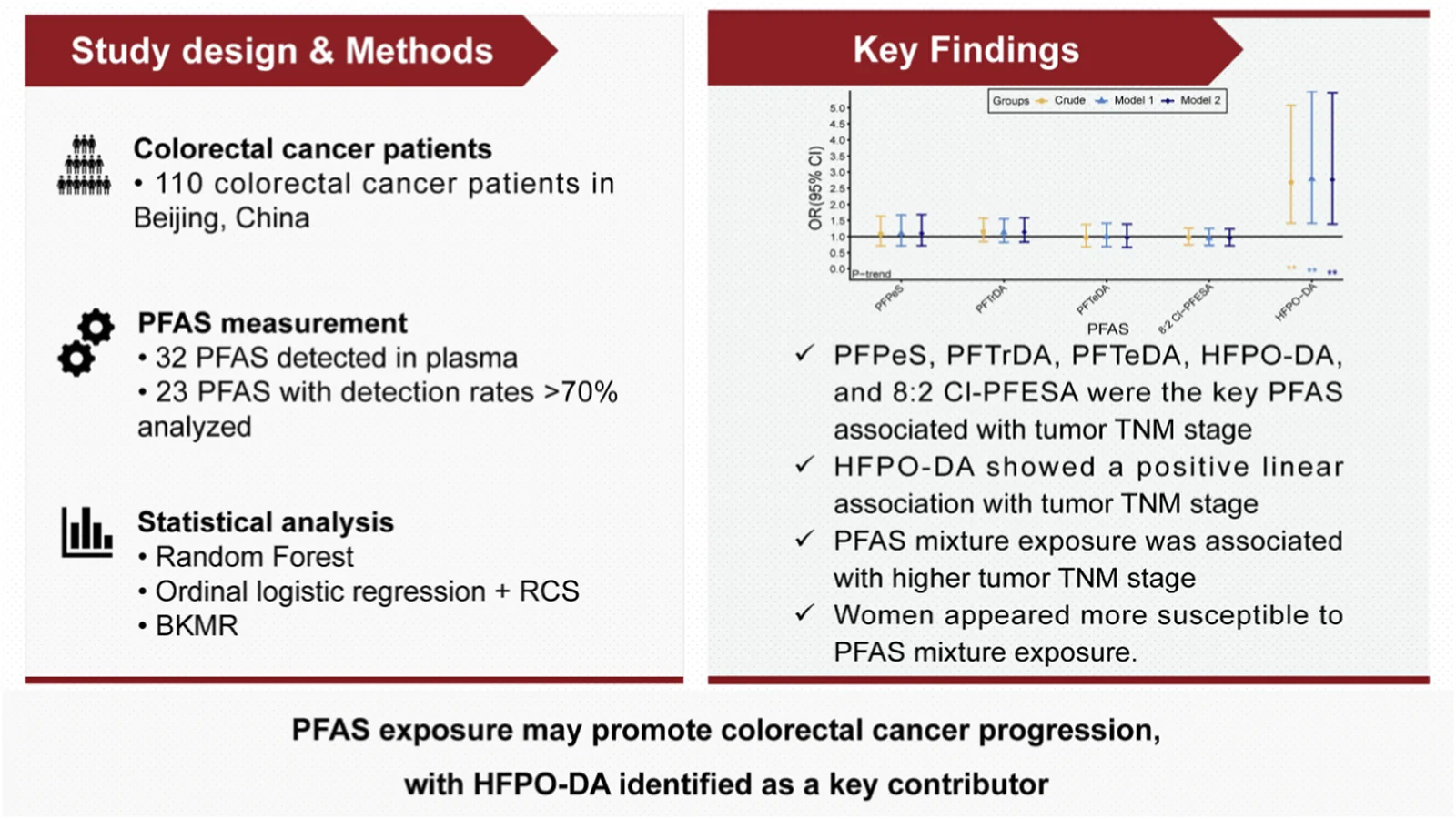

## 1 Introduction

Colorectal cancer (CRC) remains a major global health burden, with its incidence and mortality continuing to rise worldwide. Projections estimate approximately 2.2 million new cases and 1.1 million deaths annually by the end of this decade (Arnold et al., 2017; Yoshino et al., 2021). The prognosis of CRC is strongly stage-dependent (Chen et al., 2025), with 5-year survival rates declining from approximately 90% for localized disease to 71% for regional disease and 14% for distant metastatic disease (Verdina et al., 2024). The tumor-node-metastasis (TNM) staging system, which reflects primary tumor invasion, regional lymph node involvement, and distant metastasis, is therefore central to treatment decision-making and prognostic evaluation (Arrichiello et al., 2022). Beyond genetic predisposition and lifestyle factors, environmental pollutants have increasingly been implicated in CRC development and progression (Hofseth et al., 2020; Shih et al., 2023). However, most epidemiological studies have focused on cancer occurrence rather than tumor aggressiveness or clinical stage (López-Abente et al., 2012). Identifying environmental exposures associated with TNM stage may therefore provide additional evidence for understanding CRC progression and improving risk stratification.

Per- and polyfluoroalkyl substances (PFAS) are a large group of synthetic chemicals widely used in industrial and consumer products because of their thermal stability and hydrophobic and oleophobic properties (Zheng et al., 2024). Owing to their environmental persistence and bioaccumulative potential, PFAS are frequently detected in human biological samples, including blood (Liu et al., 2023). Human exposure occurs mainly through drinking water, dietary intake, and daily consumer-product use (Evich et al., 2022). PFAS exposure has been associated with multiple adverse health outcomes, including endocrine disruption, immunotoxicity, hepatotoxicity, reproductive toxicity, and carcinogenic effects (Fenton et al., 2021). The International Agency for Research on Cancer has classified perfluorooctanoic acid as carcinogenic to humans and perfluorooctanesulfonic acid as possibly carcinogenic to humans, based in part on mechanistic evidence related to oxidative stress, immune dysregulation, and epigenetic alterations (Zahm et al., 2024; Zhu et al., 2025).

The potential carcinogenicity of PFAS has been supported by experimental and epidemiological evidence. Animal studies have linked exposure to PFAS, particularly PFOA and PFOS, with Leydig cell adenomas, pancreatic acinar cell tumors, and hepatocellular adenomas or carcinomas (Durham et al., 2023; Biegel et al., 2001; Butenhoff et al., 2012). Epidemiological studies have also reported increased cancer risks among populations with occupational or environmental PFAS exposure, including firefighters, industrial workers, and residents in contaminated areas (Zheng et al., 2023; Winquist et al., 2023). For CRC specifically, emerging evidence suggests that PFAS may be associated with cancer development (Sezavar et al., 2024). Nevertheless, previous studies have largely examined cancer incidence or prevalence, while the potential role of PFAS in tumor progression remains insufficiently understood.

TNM stage provides an integrated clinical measure of tumor invasion, nodal involvement, and metastatic spread, and therefore represents a useful outcome for evaluating factors potentially related to tumor aggressiveness (Compton and Greene, 2004). Recent evidence showed that serum PFAS mixtures were associated with an increased number of metastatic lymph nodes in CRC patients, with PFOS appearing to contribute substantially to this association (Cui et al., 2024). This finding suggests that PFAS exposure may be related not only to CRC occurrence but also to disease progression. However, humans are simultaneously exposed to multiple PFAS congeners, including both legacy compounds and emerging substitutes, and single-pollutant models may not adequately reflect real-world exposure patterns. To date, evidence regarding the association between PFAS mixture exposure and TNM stage in CRC remains limited.

Therefore, this study aimed to investigate the associations of individual and mixed PFAS exposures with tumor TNM stage among patients with CRC. By integrating random forest screening, multivariable regression, restricted cubic spline analysis, and Bayesian kernel machine regression, we sought to identify key PFAS compounds and evaluate their potential joint effects on CRC progression. This study may provide preliminary epidemiological evidence for the role of PFAS exposure in CRC tumor advancement and inform future research on environmental determinants of cancer progression.

## 2 Materials and methods

### 2.1 Study population

This single-center cross-sectional observational study included 110 patients with colorectal cancer, including 49 women and 61 men, recruited from Peking University Third Hospital between March 2024 and June 2024. Patients were excluded if they had a history of previous malignancy other than colorectal cancer, severe heart failure, defined as New York Heart Association class III or above, liver cirrhosis, end-stage renal disease, autoimmune disease, other tumors, or incomplete clinical records.

Clinical and demographic data were obtained from medical records. The study was approved by the Medical Science Research Ethics Committee of Peking University Third Hospital, Beijing, China, 2022 YLS No. 554-01. All participants provided written informed consent, and all procedures involving human participants were conducted in accordance with the Declaration of Helsinki and institutional ethical standards.

### 2.2 PFAS quantification

Peripheral blood samples were collected from all participants at enrollment using anticoagulant-treated tubes. Plasma was separated by centrifugation, aliquoted into clean polypropylene tubes, and stored at −80 °C until analysis. Before PFAS quantification, samples were thawed at 4 °C.

PFAS concentrations were measured using a sensitive and validated solid-phase extraction coupled with ultra-performance liquid chromatography–tandem mass spectrometry method. Briefly, 0.1 mL of plasma was transferred to a polypropylene tube, spiked with isotope-labeled internal standards, and vortex mixed. Sample extraction was performed using an Oasis HLB cartridge, 3 mL/150 mg, Waters, Milford, MA, United States, preconditioned with 1 mL methanol and 1 mL 0.1 M formic acid. After sample loading, the cartridge was washed sequentially with 1 mL 0.1 M formic acid, 3 mL 0.1 M formic acid/methanol, 4:1, v/v, and 0.5 mL water containing 1% ammonium acetate, followed by vacuum drying for 30 min. PFAS were eluted with 1.8 mL acetonitrile containing 1% ammonium acetate. The eluate was evaporated to dryness, reconstituted in 100 μL methanol/water, 7:3, v/v, centrifuged at 15,000 rpm for 15 min, and transferred to an autosampler vial.

UPLC–MS/MS analysis was performed on an ExionLC™ AD-Triple Quad™ 6,500+ system, AB SCIEX, Framingham, MA, USA, operated in negative electrospray ionization mode. Chromatographic separation was achieved using a ZORBAX RRHD Eclipse Plus C18 column, 2.1 × 50 mm, 1.8 μm, Agilent. To ensure analytical reliability, solvent blanks, blank controls, and quality control samples at different concentration levels were analyzed every 30 samples to monitor background contamination and instrument stability. The intra-assay and inter-assay coefficients of variation for all PFAS homologues were below 10%. The detailed PFAS measurement method has been described elsewhere (Ao et al., 2023).

A total of 32 PFAS were measured. Nine compounds with detection frequencies below 70% were excluded, leaving 23 PFAS for statistical analysis. These included ten perfluoroalkane sulfonic acids, FOSA, PFBS, PFHpS, n-PFHxS, n-PFOS, PFPeS, 6m-PFOS, 3,4,5m-PFOS, 1m-PFOS, and Br-PFHxS; nine perfluoroalkyl carboxylic acids, PFOA, PFNA, PFDA, PFHxA, PFBA, PFPeA, PFTrDA, PFTeDA, and PFUnDA; and four short-chain or emerging replacement PFAS, 4:2 FTS, 6:2 Cl-PFESA, 8:2 Cl-PFESA, and HFPO-DA (Xing et al., 2025). Full names, detection frequencies, and limits of detection are provided in Supplementary Table S1. Concentrations below the limit of detection were replaced by LOD/√2 (Yu et al., 2021).

### 2.3 Colorectal cancer data

The tumor-node-metastasis staging system was used as the primary outcome variable. TNM stage reflects tumor invasion depth, regional lymph node involvement, and distant metastasis status. Tumor staging was determined according to the eighth edition of the American Joint Committee on Cancer staging criteria. Specifically, TNM stage was assigned based on postoperative histopathological findings and preoperative imaging, including computed tomography of the chest, abdomen, and pelvis. Patients were classified into Stage I, Stage II, Stage III, or Stage IV for statistical analyses.

### 2.4 Covariates

Demographic and lifestyle information was collected using a standardized questionnaire, including sex, male or female; age, years; body mass index, kg/m^2^; marital status, unmarried/divorced/widowed or married; smoking status, yes or no; and drinking status, yes or no. BMI was calculated as weight in kilograms divided by height in meters squared. Because the number of participants in some TNM-stage groups was small for marital status, this variable was excluded from the final models. The final analyses included sex, age, BMI, smoking status, and drinking status.

### 2.5 Statistical analyses

Continuous variables were expressed as mean ± standard deviation, and categorical variables as counts and percentages. Baseline characteristics were compared across TNM-stage groups using methods appropriate for variable type and distribution. One-way analysis of variance was used to compare age and BMI, and Fisher’s exact test was used for categorical variables, including sex, marital status, smoking status, drinking status, and Tumor location.

PFAS concentrations were summarized as median and interquartile range. Spearman’s rank correlation analysis was used to assess correlations among individual PFAS congeners. The Kruskal–Wallis rank-sum test was used to compare plasma concentrations of the 23 PFAS across TNM-stage groups. All PFAS concentrations were ln-transformed before analysis to improve normality. Except for the tertile-based ordinal logistic regression analyses, all subsequent analyses used ln-transformed PFAS concentrations as continuous exposure variables.

A random forest classification model was fitted with TNM stage (Stages I–IV) as a four-category outcome and all 23 ln-transformed PFAS concentrations entered simultaneously as predictors. The model was constructed using 500 trees, with four candidate predictors randomly sampled at each split (mtry = 4), and a random seed of 124 was specified to ensure reproducibility. Variable importance was quantified by the mean decrease in Gini impurity accumulated across all trees, with higher values indicating a greater contribution to TNM-stage classification. The five highest-ranking PFAS were carried forward to subsequent single-PFAS regression, restricted cubic spline, and BKMR analyses (Wang et al., 2024; Marcos-Pasero et al., 2021; Diaz-Galvan et al., 2021; Breiman, 2001).

Multivariable ordinal logistic regression models were then used to assess associations between individual PFAS exposures and TNM stage. First, ln-transformed PFAS concentrations were analyzed as continuous variables to evaluate linear trends. Second, PFAS concentrations were categorized into tertiles, Q1, Q2, and Q3, based on population exposure levels, with Q1 as the reference group. Restricted cubic spline models were further used to explore potential nonlinear exposure–response relationships between ln-transformed PFAS concentrations and TNM stage, adjusted for sex, age, and BMI (Durrleman and Simon, 1989).

Bayesian kernel machine regression models were constructed to estimate the joint effects of PFAS mixtures on TNM stage and to calculate posterior inclusion probabilities for identifying major contributing compounds (Bobb et al., 2018). Model 1 was adjusted for sex, age, and BMI. Model 2 was further adjusted for smoking status and drinking status. To assess the robustness of the findings, sex- and age-stratified BKMR analyses were performed. In addition, sex-stratified ordinal logistic regression analyses were conducted for the five selected PFAS, and PFAS-by-sex interaction terms were included to evaluate effect modification by sex.

Two sets of sensitivity analyses were conducted. First, to assess whether the results were sensitive to the variable-prioritization procedure, we repeated the ordinal logistic regression and BKMR analyses using all 23 PFAS. Second, extreme observations were defined as concentrations exceeding the third quartile plus three times the interquartile range (Q3 + 3 × IQR) on the original, untransformed scale. Compound-specific extreme values were excluded from the single-PFAS analyses, and participants with an extreme concentration in any of the five selected PFAS were excluded from the BKMR analysis.

All statistical tests were two-sided, and statistical significance was defined as P < 0.05. Analyses were performed using R software, version 4.4.1.

## 3 Results

### 3.1 Study population characteristics

Table 1 presents the baseline characteristics of patients with different TNM stages (n = 110). Among the study population, there were 61 males (55.5%) and 49 females (44.5%). The mean age was 66.3 years (SD = 11.2), and the mean BMI was 24.1 kg/m^2^ (SD = 3.6). Most participants were married (83.6%), 29.1% were smokers and 27.3% reported alcohol consumption.

**TABLE 1:** Baseline characteristics of all participants.

Characteristics	Total population	TNM stage				P^1^
	(n = 110)	I (n = 11)	II (n = 28)	III (n = 37)	IV (n = 34)	
Sex (n, %)	​	​	​	​	​	0.481
Male	61 (55.5)	4 (3.6)	18 (16.4)	20 (18.2)	19 (17.3)	​
Female	49 (44.5)	7 (6.4)	10 (9.1)	17 (15.5)	15 (13.6)	​
Age (years, mean ± SD)	66.3 ± 11.2	66.2 ± 6.05	67.8 ± 10.2	65.4 ± 10.9	66.2 ± 13.6	0.789
BMI (kg/m^2^, mean ± SD)	24.1 ± 3.6	23.8 ± 2.1	23.5 ± 3.5	24.2 ± 4.0	24.4 ± 3.7	0.806
Marriage (n, %)	​	​	​	​	​	0.199
Unmarried/Divorced/Widowed	18 (16.4)	0 (0)	6 (5.5)	4 (3.6)	8 (7.3)	​
Married	92 (83.6)	11 (10.0)	22 (20.0)	33 (30.0)	26 (23.6)	​
Smoking status (n, %)	​	​	​	​	​	0.506
No	78 (70.9)	10 (9.1)	19 (17.3)	26 (23.6)	23 (20.9)	​
Yes	32 (29.1)	1 (0.9)	9 (8.2)	11 (10.0)	11 (10.0)	​
Drinking status (n, %)	​	​	​	​	​	0.313
No	80 (72.7)	9 (8.2)	23 (20.9)	23 (20.9)	25 (22.7)	​
Yes	30 (27.3)	2 (1.8)	5 (4.5)	14 (12.7)	9 (8.2)	​
Tumor location (n, %)	​	​	​	​	​	0.137
Left-sided colorectal cancer	61 (55.5)	6 (5.5)	15 (13.6)	26 (23.6)	14 (12.7)	​
Right-sided colorectal cancer	41 (37.3)	5 (4.5)	11 (10.0)	9 (8.2)	16 (14.5)	​
Missing	8 (7.3)	0 (0)	2 (1.8)	2 (1.8)	4 (3.6)	​

^*^ Abbreviations: TNM, tumor-node-metastasis; BMI, body mass index; SD, standard deviation.

1 ANOVA, was used for Age and BMI. Fisher’s exact test was applied for Sex, Marriage, Smoking status, Drinking status, and Tumor location.

There were no statistically significant differences among the TNM stage groups in terms of sex, age, body mass index, marital status, smoking, or drinking status (all P > 0.05). Overall, the demographic and lifestyle characteristics were similar across different TNM stages.

### 3.2 PFAS concentrations and screening of key PFAS associated with tumor TNM stage

Supplementary Tables S1, S2 summarizes the distribution characteristics of PFAS in the study population. Overall, most PFAS showed high detection frequencies, with the majority exceeding 80%. Among the 23 PFAS included in subsequent analyses, nine compounds were detected in all samples (100%), namely, n-PFOS, 6m-PFOS, 3,4,5m-PFOS, 1m-PFOS, PFOA, PFNA, PFDA, PFUnDA, and 6:2 Cl-PFESA. Their geometric mean concentrations ranged from 0.214 to 3.304 ng/mL, with PFOA and n-PFOS showing the highest levels. In general, long-chain PFAS exhibited higher detection frequencies and concentration levels.

As shown in Supplementary Table S3, plasma concentrations of the 23 PFAS were generally similar across TNM stages. Median concentrations of major PFAS, including PFOA, PFOS, and PFHxS, varied only slightly among stage groups. HFPO-DA showed a higher median level in stage III–IV patients than in stage I–II patients, although the difference was not statistically significant (P = 0.070). Correlation analysis further showed significant positive correlations among most PFAS, especially within the same structural subclasses (Supplementary Figure S1). Correlation coefficients ranged from −0.24 to 0.95, with particularly strong correlations among long-chain PFCAs and among PFSAs, suggesting substantial co-exposure patterns.

**FIGURE 1:**
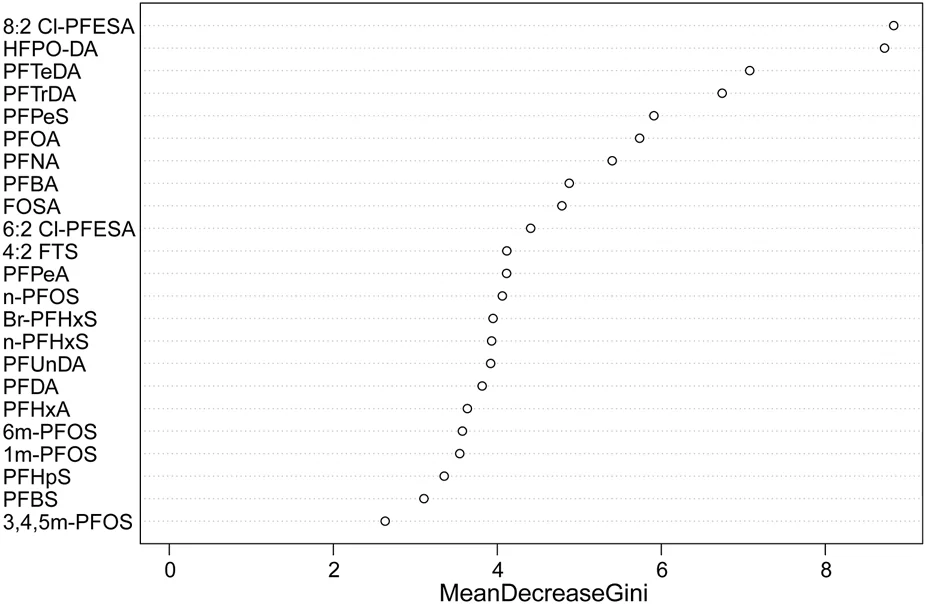
Feature selection via Random Forest Algorithm. *A random forest classification model was constructed with tumor TNM stage as the outcome and 23 PFAS as candidate predictors. Variable importance was evaluated using the Mean Decrease Gini index, with higher values indicating greater contribution to classification performance. PFAS were ranked according to their importance scores, and the five highest-ranking compounds were identified as the key PFAS for subsequent analyses. The model was fitted in R using the randomForest package with default settings (ntree = 500, mtry = 4) and a random seed of 124.

To further identify the PFAS most relevant to tumor TNM stage, a Random Forest model was established with the 23 PFAS entered simultaneously as candidate predictors. The relative importance of each PFAS was assessed using the Mean Decrease Gini index, in which a larger value reflects a greater contribution to model-based classification of TNM stage. As shown in Figure 1, 8:2 Cl-PFESA, HFPO-DA, PFTeDA, PFTrDA, and PFPeS ranked highest among the 23 PFAS, indicating that these compounds contributed more substantially than the others to the discrimination of tumor TNM stage. The importance scores of the remaining PFAS were comparatively lower, suggesting weaker contributions to model performance. Therefore, these five PFAS were considered the most informative exposure variables in relation to TNM stage and were retained as key PFAS for subsequent analyses.

### 3.3 Single pollutant analysis: PFAS congeners

Table 2 present the associations between individual plasma PFAS concentrations and TNM stage estimated using ordinal logistic regression models. When PFAS concentrations were analyzed as continuous variables, higher plasma HFPO-DA concentrations were significantly associated with more advanced TNM stage, whereas no statistically significant associations were observed for the other PFAS congeners. In the fully adjusted model, each one-unit increase in ln-transformed HFPO-DA concentration was associated with 2.76-fold higher cumulative odds of being in a more advanced TNM-stage category (95% CI: 1.39–5.46, P = 0.004).

**TABLE 2:** ORs and 95% CIs for tumor TNM stage of colorectal cancer according to PFAS exposure.

PFAS	Continuous PFAS levels		Categorical PFAS levels			
	OR (95% CI)	P	Q1	Q2	Q3	P_-trend_
*PFPeS*						
Crude	1.08 (0.71, 1.63)	0.720	Ref	1.45 (0.65, 3.23)	1.45 (0.62, 3.40)	0.373
Model 1	1.09 (0.71, 1.67)	0.689	Ref	1.43 (0.64, 3.21)	1.50 (0.63, 3.59)	0.341
Model 2	1.10 (0.72, 1.68)	0.666	Ref	1.43 (0.63, 3.21)	1.57 (0.66, 3.78)	0.296
*PFTrDA*						
Crude	1.15 (0.84, 1.57)	0.372	Ref	1.45 (0.64, 3.28)	1.18 (0.52, 2.71)	0.671
Model 1	1.13 (0.82, 1.55)	0.452	Ref	1.44 (0.63, 3.31)	1.12 (0.48, 2.59)	0.772
Model 2	1.14 (0.83, 1.58)	0.414	Ref	1.41 (0.61, 3.26)	1.12 (0.48, 2.61)	0.767
*PFTeDA*						
Crude	0.97 (0.68, 1.38)	0.850	Ref	0.62 (0.26, 1.44)	1.10 (0.49, 2.49)	0.804
Model 1	0.99 (0.69, 1.41)	0.938	Ref	0.61 (0.26, 1.42)	1.11 (0.49, 2.51)	0.789
Model 2	0.97 (0.67, 1.39)	0.849	Ref	0.61 (0.26, 1.42)	1.09 (0.48, 2.48)	0.810
*8:2 Cl-PFESA*						
Crude	0.97 (0.74, 1.26)	0.810	Ref	0.77 (0.33, 1.77)	0.60 (0.26, 1.37)	0.223
Model 1	0.95 (0.73, 1.25)	0.734	Ref	0.75 (0.32, 1.74)	0.59 (0.25, 1.36)	0.215
Model 2	0.95 (0.72, 1.24)	0.685	Ref	0.76 (0.32, 1.81)	0.57 (0.24, 1.32)	0.185
*HFPO-DA*						
Crude	2.68 (1.41, 5.08)^**^	0.003	Ref	2.02 (0.88, 4.66)^*^	2.27 (0.97, 5.34)^*^	0.059
Model 1	2.78 (1.41, 5.50)^**^	0.003	Ref	1.96 (0.84, 4.57)	2.21 (0.89, 5.54)^*^	0.079
Model 2	2.76 (1.39, 5.46)^**^	0.004	Ref	1.87 (0.80, 4.41)	2.14 (0.85, 5.38)	0.097

^*^ Odds ratios and 95% confidence intervals were estimated using ordinal logistic regression models for each PFAS. Continuous PFAS concentrations were natural logarithm-transformed before analysis. For categorical analyses PFAS concentrations were divided into tertiles with the lowest tertile (Q1) as the reference group. An OR greater than 1 indicates higher odds of being classified at a more advanced TNM stage. The crude model was unadjusted; Model 1 was adjusted for sex, age, and BMI; and Model 2 was further adjusted for smoking status and drinking status.

^*^P < 0.10.

^**^ P < 0.05.

When PFAS concentrations were analyzed by tertiles, participants in the highest HFPO-DA tertile had higher cumulative odds of being in a more advanced TNM-stage category than those in the lowest tertile, although the confidence interval included the null (OR = 2.21, 95% CI: 0.89–5.54, P = 0.089). No significant associations were observed for other PFAS in either continuous or tertile-based analyses. These findings indicate that higher plasma HFPO-DA concentrations were associated with more advanced TNM stage among patients with colorectal cancer.

Supplementary Figure S2 depicts the dose–response relationships between ln-transformed plasma PFAS concentrations and tumor TNM stage in colorectal cancer, as assessed by restricted cubic spline models. The results showed that the nonlinear trends for PFPeS, PFTrDA, PFTeDA, HFPO-DA, and 8:2 Cl-PFESA were not statistically significant (P for nonlinear >0.10). However, a significant overall trend was observed for HFPO-DA (P for overall = 0.012), exhibiting a clear linear increasing pattern. Higher plasma HFPO-DA levels were associated with an increased risk of advanced tumor TNM stage. The exposure–response curve appeared nearly linear, indicating a positive association between HFPO-DA exposure and tumor progression. These findings support a positive cross-sectional association between plasma HFPO-DA concentrations and TNM-stage severity, whereas other PFAS congeners showed no evident dose–response relationships.

### 3.4 Mixture analysis: PFAS mixtures

**FIGURE 2:**
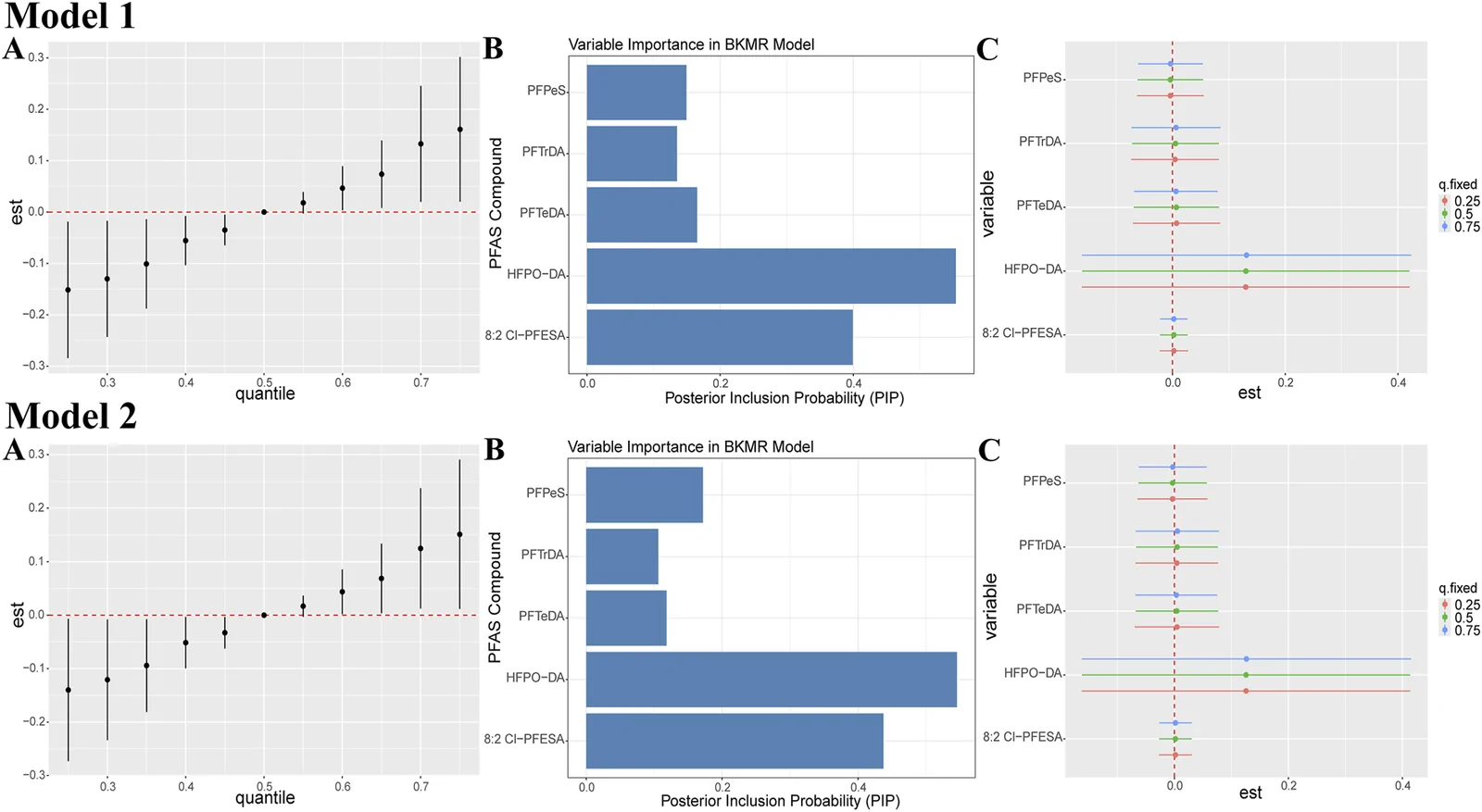
Joint effect estimates with 95% CI for different PFAS mixtures in relation to Tumor TNM Stage of Colorectal Cancer in BKMR models. *(A) Estimated overall association of the five-PFAS mixture with TNM stage when all PFAS were jointly fixed at selected exposure quantiles, relative to their median concentrations. Points represent posterior estimates, and error bars represent 95% credible intervals. **(B)** Posterior inclusion probabilities of the five PFAS in the BKMR model. **(C)** Estimated associations of individual PFAS with TNM stage when the remaining mixture components were fixed at the 25th, 50th, or 75th percentiles. Model 1 was adjusted for sex, age, and BMI; Model 2 was further adjusted for smoking status and drinking status.

The joint effects of PFAS mixture exposure on tumor TNM stage in colorectal cancer were evaluated using the BKMR model (Figure 2; Supplementary Table S4). As shown in Figure 2A, the estimated overall mixture effect increased progressively across higher PFAS mixture quantiles, suggesting a positive association between combined PFAS concentrations and more advanced TNM stage. This trend remained consistent across both models. Based on the posterior inclusion probability (PIP) results, HFPO-DA and 8:2 Cl-PFESA were identified as the major contributors to the overall mixture effect, with PIPs of 0.546 and 0.437, respectively (Figure 2B; Supplementary Table S4).

Figure 2C further shows that HFPO-DA was positively associated with TNM stage when the other mixture components were held constant, whereas the corresponding associations for the other PFAS were weaker or included the null. Collectively, these findings suggest that HFPO-DA contributed most consistently to the observed positive association between the PFAS mixture and more advanced TNM stage.

### 3.5 Stratified analysis

To examine potential sex-related differences in PFAS-associated effects, sex-stratified analyses were performed. Sex-stratified ordinal logistic regression showed that higher HFPO-DA concentrations were associated with more advanced TNM stage among men (OR = 2.93, 95% CI: 1.12–7.69), although none of the PFAS-by-sex interaction terms reached statistical significance (Supplementary Table S5). Sex-stratified BKMR analyses were subsequently conducted to evaluate the robustness of the PFAS mixture effects (Supplementary Figure S3; Supplementary Table S6). Among men, the overall mixture effect varied little across increasing quantiles of PFAS mixture exposure, with no apparent exposure–response pattern. In contrast, the overall mixture effect among women showed an increasing trend across higher quantiles of PFAS mixture exposure (Supplementary Figure S3A).

Differences in the PIP distribution were observed between sexes (Supplementary Figure S3B; Supplementary Table S6). In man, HFPO-DA (PIP = 0.476) and PFPeS (PIP = 0.445) were the main contributors to the overall mixture effect, whereas in woman, 8:2 Cl-PFESA (PIP = 0.485) and HFPO-DA (PIP = 0.311) appeared to be more influential. The individual PFAS effect estimates (Supplementary Figure S3C) were generally consistent with these patterns.

In both the ≥65 and <65-year groups, the joint effect estimates of PFAS mixtures on tumor TNM stage showed no significant differences across increasing exposure quantiles (Supplementary Figure S4; Supplementary Table S7). Overall, the association between PFAS mixture exposure and tumor TNM stage of colorectal cancer remained stable across age strata, suggesting that age had little modifying effect on this association.

### 3.6 Sensitivity analysis

In sensitivity analyses evaluating all 23 PFAS individually, HFPO-DA remained positively associated with more advanced TNM stage after covariate adjustment, whereas none of the other PFAS showed a statistically significant association (Supplementary Table S8). When all 23 PFAS were simultaneously included in the BKMR model, the estimated overall mixture association was attenuated and less precise, with the 95% credible intervals including the null across the evaluated exposure quantiles. Nevertheless, the exposure–response pattern remained directionally consistent with the primary analysis, showing a weak upward trend (Supplementary Figure S5). HFPO-DA retained the highest posterior inclusion probability, and no other PFAS showed a higher relative contribution to the mixture association (Supplementary Table S9). After excluding extreme observations, the continuous association between HFPO-DA and more advanced TNM stage remained statistically significant in the fully adjusted model (Supplementary Table S10). The corresponding five-PFAS BKMR analysis also showed an increasing exposure–response pattern consistent with the primary analysis, although the association was not statistically significant (Supplementary Figure S6; Supplementary Table S11).

## 4 Discussion

In this cross-sectional study of patients with colorectal cancer, plasma PFAS concentrations were evaluated in relation to tumor TNM stage. The main finding was that higher PFAS mixture concentrations were associated with more advanced TNM stage, with HFPO-DA showing the most consistent positive association across single-pollutant and mixture analyses. Random forest analysis identified several PFAS congeners, including HFPO-DA, 8:2 Cl-PFESA, PFTeDA, PFTrDA, and PFPeS, as the highest-ranking exposure variables related to TNM stage. In multivariable regression and restricted cubic spline analyses, HFPO-DA showed a positive and approximately linear association with more advanced TNM stage. BKMR further suggested a positive joint association between PFAS mixtures and more advanced TNM stage, with HFPO-DA and 8:2 Cl-PFESA contributing most to the overall mixture association. These findings indicate that plasma PFAS concentrations, particularly those of emerging replacement compounds, were associated with TNM-stage severity among patients with colorectal cancer.

Previous studies on PFAS and cancer have mainly focused on cancer incidence or prevalence rather than tumor characteristics after diagnosis. According to clinical guidance and the International Agency for Research on Cancer evaluation, PFAS have carcinogenic potential, although human evidence remains incomplete and varies by cancer site (National Academies of Sciences, 2022). For colorectal cancer, epidemiological findings have been inconsistent. An occupational study reported an excess of malignant colorectal tumors among workers exposed to perfluorooctanesulfonyl fluoride, whereas a cohort study of PFOA-exposed workers did not observe a clear positive association with colorectal cancer (Olsen et al., 2004; Steenland et al., 2015). A large study from PFOA-contaminated water districts even reported an inverse association of colorectal cancer prevalence with serum PFOS and, to a lesser extent, PFOA, although the cross-sectional design limited causal interpretation (Innes et al., 2014). More broadly, association between circulating PFAS concentrations and tumor development may varies across individual compounds and different cancer types. Whether these PFAS were specifically elevated in colorectal cancer patients still need to be clarified using large contemporaneously recruited comparison group. Experimental findings are also mixed; for example, chronic oral PFOS exposure reduced gastrointestinal tumor burden in APC^min^ mice, suggesting that the biological effects of PFAS may depend on exposure context, compound type, dose, and disease model (Wimsatt et al., 2016). Compared with previous studies focused primarily on cancer occurrence, the present study examines PFAS concentrations in relation to TNM-stage severity among patients with established colorectal cancer.

A notable finding of this study was the positive association between plasma HFPO-DA concentrations and more advanced TNM stage. HFPO-DA is a short-chain PFAS substitute introduced as an alternative to legacy long-chain PFAS. Although such substitutes are often considered to have lower bioaccumulation potential, increasing evidence suggests that they may still have biological activity and potential toxicity (Guo et al., 2021). In the present analyses, legacy PFAS, including PFOA, PFOS, and PFNA, were frequently detected but were not significantly associated with TNM stage, whereas HFPO-DA showed the most consistent positive association across the single-PFAS analyses. This finding does not establish causality, but it raises concern that emerging PFAS substitutes may not be risk-free and should be considered in future environmental cancer research and risk assessment.

Several biological pathways identified in experimental studies may provide biological context for the observed association between PFAS concentrations and TNM-stage severity. *In vitro* studies have shown that PFOS and PFOA can increase the migratory capacity of three-dimensional colorectal cancer organoids in a dose- and time-dependent manner (Zheng et al., 2023). Mechanistic analyses further suggest that PFAS exposure may promote epithelial–mesenchymal transition, as indicated by increased N-cadherin and vimentin expression and decreased E-cadherin expression (Li et al., 2025). These changes are closely related to tumor invasion and metastasis. PFAS may also influence metabolic and inflammatory pathways relevant to tumor progression. Activation of PPAR α/γ signaling and disruption of AKT/mTOR pathways may alter lipid and bile acid metabolism and contribute to insulin resistance (Zhu et al., 2025), while metabolomics studies have linked PFAS exposure to changes in fatty acid metabolism (Chen et al., 2020). In addition, PFOS and PFOA have been reported to affect pathways involved in cell proliferation, apoptosis, endocrine regulation, and immune function (Sunderland et al., 2019). PFAS may also interact with detoxification-related proteins such as GSTA1, potentially impairing cellular defense against oxidative and electrophilic stress (Li et al., 2025). Moreover, PFOS has been shown to activate the PI3K/Akt–NF-κB pathway, which may further contribute to epithelial–mesenchymal transition and tumor invasiveness (Li et al., 2024). Together, these mechanisms provide biological plausibility for an association between PFAS exposure and more advanced tumor stage, although direct experimental evidence for HFPO-DA in colorectal cancer remains limited.

The sex-stratified analysis suggested that the association between PFAS mixtures and TNM stage was more pronounced among women, whereas age-stratified analyses showed no clear effect modification. Sex-specific associations have also been reported in previous PFAS studies. For example, higher serum PFOA concentrations have been associated with increased kidney cancer risk among women but not men (Winquist et al., 2023). Analyses of NHANES data also reported PFAS-related cancer associations that were more evident among women for certain cancer types, including melanoma (Cathey et al., 2023). These differences may reflect sex-related variation in PFAS toxicokinetics, hormonal regulation, lipid distribution, renal clearance, and elimination half-lives (Roth et al., 2021; Li et al., 2022; Xu et al., 2020). However, because the present stratified analyses were based on a limited sample size, the sex-specific findings should be interpreted cautiously and considered exploratory. Larger studies are needed to determine whether sex modifies the relationship between PFAS exposure and colorectal cancer progression.

This study has several strengths. First, it focused on TNM stage, a clinically meaningful indicator of tumor invasion, nodal involvement, and metastasis, rather than only cancer occurrence. Second, it measured a broad panel of PFAS congeners, including both legacy compounds and emerging substitutes. Third, multiple analytical approaches were applied, including random forest screening, multivariable regression, restricted cubic spline analysis, and BKMR, allowing assessment of both individual compounds and mixture effects. These approaches provide complementary evidence and help address the complexity of real-world PFAS co-exposure.

Several limitations should also be considered. First, the cross-sectional design precludes causal inference and cannot establish whether PFAS exposure preceded tumor progression. Reverse causation is also possible, because disease status, treatment, diet, or metabolic changes may influence circulating PFAS concentrations. Moreover, because most blood samples were collected within 1 year before or after treatment, post-treatment changes in nutritional status, serum protein binding, renal or hepatic function, body composition, and fluid balance may have influenced PFAS concentrations in some participants. Nevertheless, the relatively slow elimination of many PFAS suggests that the measured concentrations likely retained information on pre-existing body burden (Xu et al., 2020; Li et al., 2018; Louisse et al., 2023). Second, the sample size was relatively small, especially in stratified analyses, which may limit statistical power and increase uncertainty in effect estimates. Third, although several demographic and lifestyle covariates were adjusted for, residual confounding by diet, occupation, socioeconomic factors, family history, genetic susceptibility, and other environmental exposures cannot be excluded. Fourth, plasma PFAS concentrations reflect internal exposure at a single time point and may not fully capture long-term cumulative exposure, early-life exposure, or tissue-specific accumulation. Finally, the biological mechanisms underlying the observed association, particularly for HFPO-DA and other emerging PFAS substitutes, require further validation in cell, animal, and longitudinal epidemiological studies.

## 5 Conclusion

In conclusion, higher plasma HFPO-DA concentrations were associated with more advanced TNM stage among patients with colorectal cancer, and BKMR showed a concordant increasing pattern for the overall PFAS mixture. HFPO-DA also showed the highest relative contribution to the mixture association. These cross-sectional findings identify HFPO-DA as an emerging PFAS warranting further investigation in relation to colorectal cancer stage severity, but they do not establish temporality or causality. Larger prospective studies with pretreatment and repeated PFAS measurements are needed to confirm these associations.

## Data Availability

The raw data supporting the conclusions of this article will be made available by the authors, without undue reservation.
