# Supplementary material for: Per- and polyfluoroalkyl substances mixture exposure is associated with colorectal cancer TNM stage
Source: Front Toxicol. 2026 Aug 11;8:1904192. doi: 10.3389/ftox.2026.1904192 (PMC13504314; doi:10.3389/ftox.2026.1904192)
Supplement: Supplementary file 1 [file Supplementaryfile1.docx]

**Supplementary materials**

**Per- and polyfluoroalkyl substances mixture exposure is associated with colorectal cancer TNM stage**

Ning Kang^1,2#^, Yang Zhao^3#^, Zhi Huang^4,5#^, Rongbin Li^4,5^, Xiaoqian Ma^4,5^, Shigang Ding^4,5^, Fei Li^6*^, Shengju Yin^1,2*^, Weiwei Fu^4,5*^

^#^These authors contributed equally to the work.

**Affiliation**

^1^Department of Maternal and child health, School of Public Health, Shanghai Jiao Tong University School of Medicine, Shanghai, 200025, China.

^2^Ministry of Education-Shanghai Key Laboratory of Children’s Environmental Health, Xinhua Hospital, Shanghai Jiao Tong University School of Medicine, Shanghai, 200025, China.

^3^Department of Laboratory Medicine, Peking University Third Hospital, Beijing, China.

^4^Department of Gastroenterology, Peking University Third Hospital, Beijing, China.

^5^Beijing Key Laboratory for Helicobacter Pylori Infection and Upper Gastrointestinal Diseases, Beijing, China.

^6^Ministry of Education-Shanghai Key Laboratory of Children’s Environmental Health & Department of Developmental and Behavioural Paediatric & Child Primary Care, Xinhua Hospital Affiliated to Shanghai Jiao Tong University School of Medicine, 1665 Kongjiang Road, Shanghai, 200092 China.

**^*^Corresponding authors:**

Weiwei Fu, Department of Gastroenterology, Peking University Third Hospital, Beijing, China. E-mail: fuweiwei@bjmu.edu.cn

Shengju Yin, Department of Maternal and child health, School of Public Health, Shanghai Jiao Tong University School of Medicine, Shanghai, 200025, China. E-mail: yinshengju@163.com

Fei Li, Ministry of Education-Shanghai Key Laboratory of Children’s Environmental Health & Department of Developmental and Behavioural Paediatric & Child Primary Care, Xinhua Hospital Affiliated to Shanghai Jiao Tong University School of Medicine, 1665 Kongjiang Road, Shanghai, 200092 China. E-mail: feili@shsmu.edu.cn

**Contents**

Table S1. Distribution of 32 PFAS in plasma.

Table S2. Sex-specific distributions of the 23 plasma PFAS concentrations among patients with colorectal cancer.

Table S3. Serum PFAS levels by TNM stage in colorectal cancer

Table S4. PIP for each PFAS in different PFAS mixtures in BKMR models.

Table S5. Sex-stratified associations between the five selected PFAS and TNM stage in colorectal cancer.

Table S6. Subgroup analysis: Posterior inclusion probability for each PFAS in the overall mixture in BKMR models stratified by sex.

Table S7. Subgroup analysis: Posterior inclusion probability for each PFAS in the overall mixture in BKMR models stratified by age.

Table S8. Sensitivity analysis of the associations between all 23 individual PFAS and TNM stage in colorectal cancer.

Table S9. Posterior inclusion probabilities of all 23 PFAS in the sensitivity BKMR analysis of TNM stage in colorectal cancer.

Table S10. Sensitivity analysis of the associations between the five selected PFAS and TNM stage in colorectal cancer after excluding extreme observations (N=88).

Table S11. Posterior inclusion probabilities of the five PFAS in BKMR models after excluding extreme observations (N=88).

Figure S1. Spearman correlation of plasma PFAS.

Figure S2. Association between ln-transformed PFAS levels and Tumor TNM Stage of Colorectal Cancer analyzed using Restricted Cubic Spline Models.

Figure S3. Subgroup analysis: Joint effect estimates with 95% CI for the overall mixture in relation to Tumor TNM Stage of Colorectal Cancer in BKMR models stratified by sex.

Figure S4. Subgroup analysis: Joint effect estimates with 95% CI for the overall mixture in relation to Tumor TNM Stage of Colorectal Cancer in BKMR models stratified by age.

Figure S5. Sensitivity analysis of the association between the 23-PFAS mixture and TNM stage in colorectal cancer using BKMR.

Figure S6. Sensitivity analysis of the association between the five-PFAS mixture and TNM stage in colorectal cancer after excluding extreme observations using BKMR (N=88).

**Table S1. Distribution of 32 PFAS in plasma.**

| Abbreviation | Full name | LOD | LOD/√2 | >LOD (%) | GM | Percentile | | | | |
| --- | --- | --- | --- | --- | --- | --- | --- | --- | --- | --- |
|  |  |  |  |  |  | Min | Max | 25th | 50th | 75th |
| ***PFSA*** |  |  |  |  |  |  |  |  |  |  |
| FOSA | Perfluorooctane sulfonamide | 0.001 | 0.001 | 90.9 | 0.006 | <LOD | 0.072 | 0.003 | 0.005 | 0.014 |
| PFBS | Perfluorobutane sulfonic acid | 0.005 | 0.004 | 77.3 | 0.041 | <LOD | 31.222 | 0.020 | 0.059 | 0.092 |
| PFHpS | Perfluoroheptane sulfonic acid | 0.001 | 0.000 | 97.3 | 0.062 | <LOD | 0.991 | 0.031 | 0.071 | 0.150 |
| n- PFHxS | linear Perfluorohexane sulfonic acid | 0.004 | 0.002 | 99.1 | 0.452 | <LOD | 5.023 | 0.266 | 0.506 | 0.898 |
| n-PFOS | linear Perfluorooctane sulfonic acid | 0.004 | 0.002 | 100.0 | 2.313 | 0.178 | 34.141 | 1.229 | 2.444 | 4.370 |
| PFPeS | Perfluoropentane sulfonic acid | 0.001 | 0.001 | 97.3 | 0.005 | <LOD | 0.140 | 0.003 | 0.005 | 0.007 |
| 6m-PFOS | 6-methyl-Perfluorooctane sulfonic acid | 0.005 | 0.004 | 100.0 | 0.214 | 0.023 | 2.290 | 0.121 | 0.217 | 0.388 |
| 3,4,5m-PFOS | 3-/4-/5-methyl-Perfluorooctane sulfonic acid | 0.009 | 0.006 | 100.0 | 0.617 | 0.038 | 6.133 | 0.31 | 0.597 | 1.342 |
| 1m-PFOS | 1-methyl-Perfluorooctane sulfonic acid | 0.001 | 0.001 | 100.0 | 0.134 | 0.006 | 1.357 | 0.064 | 0.124 | 0.321 |
| Br- PFHxS | branched Perfluorohexane sulfonic acid | 0.009 | 0.007 | 80.0 | 0.036 | <LOD | 0.824 | 0.016 | 0.043 | 0.078 |
| PFDS^†^ | Perfluorodecane sulfonic acid | 0.006 | 0.004 | 16.4 | 0.005 | <LOD | 0.101 | <LOD | <LOD | <LOD |
| PFNS^†^ | Perfluorononane sulfonic acid | 0.010 | 0.007 | 8.2 | 0.008 | <LOD | 0.082 | <LOD | <LOD | <LOD |
| ***PFCA*** |  |  |  |  |  |  |  |  |  |  |
| PFOA | Perfluorooctanoic acid | 0.005 | 0.003 | 100.0 | 3.304 | 0.129 | 93.766 | 1.771 | 2.899 | 5.625 |
| PFNA | Perfluorononanoic acid | 0.005 | 0.004 | 100.0 | 0.522 | 0.038 | 9.476 | 0.316 | 0.469 | 0.848 |
| PFDA | Perfluorodecanoic acid | 0.006 | 0.004 | 100.0 | 0.271 | 0.018 | 6.109 | 0.147 | 0.241 | 0.488 |
| PFHxA | Perfluorohexanoic acid | 0.002 | 0.001 | 78.2 | 0.006 | <LOD | 0.176 | 0.003 | 0.007 | 0.010 |
| PFBA | Perfluorobutanoic acid | 0.003 | 0.002 | 77.3 | 0.032 | <LOD | 0.519 | 0.028 | 0.055 | 0.096 |
| PFPeA | Perfluoropentanoic acid | 0.002 | 0.001 | 71.8 | 0.005 | <LOD | 0.145 | <LOD | 0.007 | 0.010 |
| PFTrDA | Perfluorotridecanoic acid | 0.010 | 0.007 | 93.6 | 0.052 | <LOD | 1.112 | 0.027 | 0.047 | 0.102 |
| PFTeDA | Perfluorotetradecanoic acid | 0.009 | 0.006 | 96.4 | 0.105 | <LOD | 1.852 | 0.081 | 0.127 | 0.165 |
| PFUnDA | Perfluoroundecanoic acid | 0.010 | 0.007 | 100.0 | 0.250 | 0.011 | 4.427 | 0.133 | 0.239 | 0.508 |
| PFHpA^†^ | Perfluoroheptanoic acid | 0.002 | 0.001 | 59.1 | 0.007 | <LOD | 3.925 | <LOD | <LOD | 0.032 |
| PFDoA^†^ | Perfluorododecanoic acid | 0.015 | 0.011 | 40.9 | 0.024 | <LOD | 0.379 | <LOD | 0.021 | 0.041 |
| ***Short-Chain and Emerging Replacement PFAS*** | | | | |  |  |  |  |  |  |
| 4:2 FTS | 4:2 Fluorotelomer sulfonic acid | 0.001 | 0.001 | 71.8 | 0.001 | <LOD | 0.071 | <LOD | 0.002 | 0.002 |
| 6:2 Cl-PFESA | 6:2 chlorinated perfluoroalkyl ether sulfonic acid | 0.001 | 0.001 | 100.0 | 0.333 | 0.013 | 22.675 | 0.170 | 0.322 | 0.654 |
| 8:2 Cl-PFESA | 8:2 chlorinated perfluoroalkyl ether sulfonic acid | 0.000 | 0.000 | 93.6 | 0.004 | <LOD | 0.703 | 0.003 | 0.004 | 0.006 |
| HFPO-DA | Hexafluoropropylene oxide dimer acid | 0.001 | 0.001 | 100.0 | 0.008 | 0.001 | 0.033 | 0.006 | 0.008 | 0.011 |
| 6:2 FTS^†^ | 6:2 Fluorotelomer sulfonic acid | 0.003 | 0.002 | 19.1 | 0.003 | <LOD | 0.181 | <LOD | <LOD | <LOD |
| 8:2 FTS^†^ | 8:2 Fluorotelomer sulfonic acid | 0.004 | 0.003 | 2.7 | 0.003 | <LOD | 0.116 | <LOD | <LOD | <LOD |
| N-ETFOSAA^†^ | N-ethyl perfluorooctane sulfonamidoacetic acid | 0.004 | 0.003 | 2.7 | 0.003 | <LOD | 0.015 | <LOD | <LOD | <LOD |
| N-MEFOSAA^†^ | N-methyl perfluorooctane sulfonamidoacetic acid | 0.002 | 0.001 | 10.9 | 0.002 | <LOD | 0.023 | <LOD | <LOD | <LOD |
| ADONA^†^ | 4,8-dioxa-3H-perfluorononanoic acid | 0.000 | 0.000 | 30.0 | 0.000 | <LOD | 0.012 | <LOD | <LOD | 0.001 |

^*^A total of 32 PFAS were measured. Twenty-three PFAS with detection frequencies greater than 70% were included in the statistical analyses. Nine PFAS with detection frequencies below 70%, marked with a dagger (^†^), were excluded from subsequent analyses. PFAS, per- and polyfluoroalkyl substances; GM, geometric mean; LOD, limit of detection.

**Table S2. Sex-specific distributions of the 23 plasma PFAS concentrations among patients with colorectal cancer.**

|  | Median (Q1, Q3) | |
| --- | --- | --- |
|  | Men (n = 61) | Women (n = 49) |
| ***PFSA*** |  |  |
| FOSA | 0.004 (0.002, 0.011) | 0.006 (0.004, 0.020) |
| PFBS | 0.049 (0.004, 0.082) | 0.062 (0.034, 0.113) |
| PFHpS | 0.070 (0.026, 0.160) | 0.071 (0.039, 0.142) |
| n- PFHxS | 0.535 (0.301, 0.868) | 0.492 (0.264, 0.913) |
| n-PFOS | 2.366 (1.225, 4.302) | 2.473 (1.267, 4.408) |
| PFPeS | 0.004 (0.003, 0.006) | 0.005 (0.003, 0.008) |
| 6m-PFOS | 0.221 (0.121, 0.375) | 0.212 (0.121, 0.416) |
| 3,4,5m-PF0S | 0.690 (0.316, 1.480) | 0.590 (0.303, 1.190) |
| 1m-PFOS | 0.126 (0.075, 0.324) | 0.122 (0.060, 0.319) |
| Br- PFHxS | 0.041 (0.010, 0.056) | 0.053 (0.029, 0.088) |
| ***PFCA*** |  |  |
| PFOA | 2.734 (1.818, 5.288) | 3.206 (1.691, 6.699) |
| PFNA | 0.481 (0.298, 0.860) | 0.467 (0.343, 0.814) |
| PFDA | 0.235 (0.142, 0.467) | 0.273 (0.152, 0.517) |
| PFHxA | 0.006 (0.003, 0.009) | 0.007 (0.005, 0.012) |
| PFBA | 0.057 (0.026, 0.114) | 0.054 (0.035, 0.087) |
| PFPeA | 0.006 (0.001, 0.010) | 0.007 (0.001, 0.009) |
| PFTrDA | 0.039 (0.025, 0.102) | 0.064 (0.031, 0.103) |
| PFTeDA | 0.119 (0.073, 0.165) | 0.129 (0.090, 0.166) |
| PFUnDA | 0.222 (0.117, 0.481) | 0.289 (0.158, 0.552) |
| ***Short-Chain and Emerging Replacement PFAS*** | |  |
| 4:2 FTS | 0.002 (0.001, 0.002) | 0.002 (0.001, 0.002) |
| 6:2 Cl-PFESA | 0.278 (0.154, 0.525) | 0.394 (0.212, 0.751) |
| 8:2 Cl-PFESA | 0.003 (0.003, 0.005) | 0.004 (0.003, 0.008) |
| HFPO-DA | 0.008 (0.006, 0.010) | 0.008 (0.006, 0.012) |

^*^Plasma PFAS concentrations are presented as medians and interquartile ranges in ng/mL. IQR, interquartile range; PFAS, per- and polyfluoroalkyl substances.

**Table S3. Serum PFAS levels by TNM stage in colorectal cancer**

|  |  | TNM stage ^a^ | |  | P ^b^ |
| --- | --- | --- | --- | --- | --- |
|  | Ⅰ (n = 11) | Ⅱ (n = 28) | Ⅲ (n = 37) | Ⅳ (n = 34) |  |
| ***PFSA*** |  |  |  |  |  |
| FOSA | 0.004 (0.003, 0.006) | 0.004 (0.003, 0.009) | 0.006 (0.003, 0.020) | 0.006 (0.003, 0.015) | 0.484 |
| PFBS | 0.041 (0.032, 0.084) | 0.055 (0.036, 0.092) | 0.061 (0.006, 0.088) | 0.062 (0.004, 0.090) | 0.971 |
| PFHpS | 0.090 (0.046, 0.162) | 0.077 (0.037, 0.122) | 0.063 (0.029, 0.150) | 0.073 (0.027, 0.187) | 0.847 |
| n- PFHxS | 0.563 (0.377, 0.909) | 0.588 (0.350, 0.917) | 0.435 (0.189, 1.110) | 0.498 (0.181, 0.797) | 0.634 |
| n-PFOS | 2.513 (1.687, 3.944) | 1.988 (1.012, 3.478) | 2.659 (1.209, 4.685) | 2.506 (1.236, 4.965) | 0.642 |
| PFPeS | 0.005 (0.003, 0.009) | 0.005 (0.003, 0.008) | 0.004 (0.003, 0.006) | 0.005 (0.004, 0.007) | 0.421 |
| 6m-PFOS | 0.204 (0.175, 0.338) | 0.201 (0.103, 0.268) | 0.249 (0.105, 0.393) | 0.224 (0.122, 0.414) | 0.679 |
| 3,4,5m-PFOS | 0.870 (0.375, 1.473) | 0.597 (0.329, 1.029) | 0.575 (0.282, 1.480) | 0.541 (0.305, 1.176) | 0.844 |
| 1m-PFOS | 0.218 (0.076, 0.305) | 0.143 (0.087, 0.322) | 0.109 (0.052, 0.324) | 0.125 (0.058, 0.295) | 0.799 |
| Br- PFHxS | 0.035 (0.023, 0.117) | 0.046 (0.029, 0.094) | 0.043 (0.007, 0.083) | 0.036 (0.017, 0.067) | 0.592 |
| ***PFCA*** |  |  |  |  |  |
| PFOA | 3.645 (2.428, 4.351) | 3.674 (1.883, 5.073) | 2.345 (1.584, 6.259) | 2.793 (1.608, 5.378) | 0.699 |
| PFNA | 0.614 (0.454, 0.766) | 0.351 (0.250, 0.695) | 0.540 (0.322, 1.050) | 0.467 (0.335, 0.797) | 0.255 |
| PFDA | 0.278 (0.155, 0.466) | 0.200 (0.126, 0.492) | 0.249 (0.152, 0.553) | 0.251 (0.192, 0.420) | 0.590 |
| PFHxA | 0.006 (0.005, 0.008) | 0.007 (0.003, 0.009) | 0.007 (0.001, 0.009) | 0.007 (0.005, 0.013) | 0.799 |
| PFBA | 0.045 (0.028, 0.097) | 0.057 (0.036, 0.114) | 0.044 (0.002, 0.076) | 0.076 (0.039, 0.103) | 0.307 |
| PFPeA | 0.007 (0.005, 0.008) | 0.007 (0.004, 0.009) | 0.006 (0.001, 0.009) | 0.007 (0.001, 0.010) | 0.770 |
| PFTrDA | 0.064 (0.045, 0.103) | 0.032 (0.023, 0.093) | 0.046 (0.025, 0.103) | 0.061 (0.030, 0.115) | 0.337 |
| PFTeDA | 0.127 (0.099, 0.133) | 0.131 (0.077, 0.179) | 0.128 (0.075, 0.166) | 0.126 (0.079, 0.167) | 0.899 |
| PFUnDA | 0.311 (0.167, 0.504) | 0.182 (0.098, 0.464) | 0.249 (0.135, 0.517) | 0.264 (0.160, 0.520) | 0.581 |
| ***Short-Chain and Emerging Replacement PFAS*** | | |  |  |  |
| 4:2 FTS | 0.001 (0.001, 0.002) | 0.002 (0.001, 0.003) | 0.001 (0.001, 0.002) | 0.002 (0.001, 0.002) | 0.195 |
| 6:2 Cl-PFESA | 0.286 (0.219, 0.902) | 0.284 (0.136, 0.599) | 0.357 (0.129, 0.719) | 0.315 (0.213, 0.547) | 0.762 |
| 8:2 Cl-PFESA | 0.004 (0.004, 0.013) | 0.004 (0.002, 0.006) | 0.004 (0.003, 0.008) | 0.003 (0.002, 0.005) | 0.443 |
| HFPO-DA | 0.008 (0.003, 0.011) | 0.007 (0.005, 0.009) | 0.009 (0.007, 0.012) | 0.009 (0.006, 0.012) | 0.058 |

^a^ Data are presented as P50 (P25, P75).

^b^ The Kruskal-Wallis test was used for group comparisons.

**Table S4. PIP for each PFAS in different PFAS mixtures in BKMR models.**

|  | PIP | |
| --- | --- | --- |
|  | Model 1 | Model 2 |
| PFPeS | 0.149 | 0.172 |
| PFTrDA | 0.135 | 0.106 |
| PFTeDA | 0.165 | 0.118 |
| HFPO-DA | 0.554 | 0.546 |
| 8:2 Cl-PFESA | 0.399 | 0.437 |

^*^Model 1 was adjusted for sex, age, and BMI; Model 2 was further adjusted for smoking status and drinking status. Higher posterior inclusion probabilities indicate greater relative contributions to the overall mixture association.

**Table S5. Sex-stratified associations between the five selected PFAS and TNM stage in colorectal cancer.**

| PFAS | Continuous PFAS levels | | |  | Categorical PFAS levels | | | | |
| --- | --- | --- | --- | --- | --- | --- | --- | --- | --- |
|  | OR (95% CI) | P | P_-interaction_ |  | Q1 | Q2 | Q3 | P_-trend_ | P_-interaction_ |
| ***PFPeS*** |  |  | 0.522 |  |  |  |  |  | 0.062 |
| Men | 0.91 (0.50, 1.66) | 0.762 |  |  | Ref | 2.17 (0.73, 6.46) | 0.73 (0.21, 2.51) | 0.889 |  |
| Women | 1.15 (0.59, 2.26) | 0.685 |  |  |  | 0.94 (0.25, 3.47) | 2.56 (0.66, 10.00) | 0.163 |  |
| ***PFTrDA*** |  |  | 0.639 |  |  |  |  |  | 0.641 |
| Men | 1.24 (0.78, 1.96) | 0.366 |  |  | Ref | 2.26 (0.74, 6.92) | 1.20 (0.35, 4.18) | 0.6 |  |
| Women | 0.91 (0.54, 1.53) | 0.722 |  |  |  | 0.78 (0.21, 2.85) | 0.66 (0.18, 2.40) | 0.53 |  |
| ***PFTeDA*** |  |  | 0.416 |  |  |  |  |  | 0.124 |
| Men | 0.83 (0.47, 1.46) | 0.513 |  |  | Ref | 1.14 (0.36, 3.62) | 0.95 (0.32, 2.82) | 0.921 |  |
| Women | 1.15 (0.67, 1.96) | 0.611 |  |  |  | 0.36 (0.09, 1.35) | 1.39 (0.38, 5.12) | 0.622 |  |
| ***8:2 Cl-PFESA*** |  |  | 0.879 |  |  |  |  |  | 0.729 |
| Men | 0.97 (0.64, 1.47) | 0.881 |  |  | Ref | 0.54 (0.17, 1.71) | 0.51 (0.16, 1.65) | 0.248 |  |
| Women | 0.88 (0.60, 1.27) | 0.481 |  |  |  | 1.23 (0.30, 5.10) | 0.76 (0.21, 2.75) | 0.644 |  |
| ***HFPO-DA*** |  |  | 0.824 |  |  |  |  |  | 0.998 |
| Men | 2.93 (1.12, 7.69)^**^ | 0.029 |  |  | Ref | 1.82 (0.58, 5.77) | 2.40 (0.60, 9.51) | 0.197 |  |
| Women | 2.09 (0.73, 6.01) | 0.169 |  |  |  | 1.79 (0.47, 6.90) | 1.34 (0.36, 4.94) | 0.662 |  |

^*^Associations were estimated using sex-stratified ordinal logistic regression models among 61 men and 49 women. PFAS concentrations were analyzed as continuous natural logarithm-transformed variables and as tertiles defined using the exposure distributions of the full study population, with Q1 as the reference category. Models were adjusted for age, BMI, smoking status, and drinking status. P values for continuous-exposure interaction were obtained from fully adjusted models containing an ln(PFAS)-by-sex interaction term. P values for categorical-exposure interaction were obtained from global likelihood-ratio tests comparing models with and without the PFAS-tertile-by-sex interaction terms. An OR greater than 1 indicates higher cumulative odds of being classified in a more advanced TNM-stage category. ^*^ P < 0.10; ^**^ P < 0.05.

**Table S6. Subgroup analysis: Posterior inclusion probability for each PFAS in the overall mixture in BKMR models stratified by sex.**

|  | PIP | |
| --- | --- | --- |
|  | Man | Woman |
| PFPeS | 0.445 | 0.231 |
| PFTrDA | 0.284 | 0.191 |
| PFTeDA | 0.315 | 0.233 |
| HFPO-DA | 0.476 | 0.311 |
| 8:2 Cl-PFESA | 0.329 | 0.485 |

^*^Models were adjusted for age and BMI. Higher posterior inclusion probabilities indicate greater relative contributions of individual PFAS to the overall mixture association within each sex stratum.

**Table S7. Subgroup analysis: Posterior inclusion probability for each PFAS in the overall mixture in BKMR models stratified by age.**

|  | PIP | |
| --- | --- | --- |
|  | Age≥65 | Age<65 |
| PFPeS | 0.288 | 0.333 |
| PFTrDA | 0.252 | 0.253 |
| PFTeDA | 0.247 | 0.272 |
| HFPO-DA | 0.398 | 0.309 |
| 8:2 Cl-PFESA | 0.263 | 0.770 |

^*^Models were adjusted for sex and BMI. Higher posterior inclusion probabilities indicate greater relative contributions of individual PFAS to the overall mixture association within each age stratum.

**Table S8. Sensitivity analysis of the associations between all 23 individual PFAS and TNM stage in colorectal cancer.**

|  | Continuous PFAS levels | | Categorical PFAS levels | | | |
| --- | --- | --- | --- | --- | --- | --- |
|  | OR (95% CI) | P | Q1 | Q2 | Q3 | P_-trend_ |
| ***PFSA*** |  |  |  |  |  |  |
| *FOSA* |  |  |  |  |  |  |
| Crude | 1.15 (0.86, 1.52) | 0.345 | Ref | 0.80 (0.35, 1.86) | 1.40 (0.61, 3.21) | 0.418 |
| Model 1 | 1.16 (0.86, 1.55) | 0.341 | Ref | 0.82 (0.34, 1.98) | 1.43 (0.60, 3.40) | 0.384 |
| Model 2 | 1.16 (0.86, 1.57) | 0.330 | Ref | 0.80 (0.33, 1.94) | 1.43 (0.59, 3.45) | 0.378 |
| *PFBS* |  |  |  |  |  |  |
| Crude | 0.95 (0.79, 1.15) | 0.619 | Ref | 1.04 (0.45, 2.38) | 0.82 (0.36, 1.90) | 0.644 |
| Model 1 | 0.96 (0.79, 1.17) | 0.699 | Ref | 1.06 (0.46, 2.45) | 0.86 (0.36, 2.01) | 0.727 |
| Model 2 | 0.96 (0.79, 1.17) | 0.680 | Ref | 1.05 (0.45, 2.42) | 0.84 (0.36, 1.97) | 0.686 |
| *PFHpS* |  |  |  |  |  |  |
| Crude | 0.91 (0.70, 1.17) | 0.467 | Ref | 0.44 (0.19, 1.02) | 0.83 (0.36, 1.90) | 0.623 |
| Model 1 | 0.90 (0.69, 1.18) | 0.435 | Ref | 0.37 (0.16, 0.90)^**^ | 0.84 (0.36, 1.96) | 0.644 |
| Model 2 | 0.90 (0.69, 1.18) | 0.466 | Ref | 0.39 (0.16, 0.94)^**^ | 0.85 (0.36, 1.99) | 0.678 |
| *n-PFHxS* |  |  |  |  |  |  |
| Crude | 0.84 (0.62, 1.14) | 0.259 | Ref | 0.54 (0.23, 1.25) | 0.47 (0.20, 1.10)^*^ | 0.082 |
| Model 1 | 0.83 (0.61, 1.13) | 0.243 | Ref | 0.54 (0.23, 1.25) | 0.47 (0.20, 1.09)^*^ | 0.080 |
| Model 2 | 0.84 (0.62, 1.15) | 0.278 | Ref | 0.56 (0.24, 1.32) | 0.48 (0.20, 1.13)^*^ | 0.093 |
| *n-PFOS* |  |  |  |  |  |  |
| Crude | 1.13 (0.82, 1.56) | 0.453 | Ref | 0.73 (0.32, 1.66) | 1.05 (0.46, 2.40) | 0.928 |
| Model 1 | 1.11 (0.80, 1.55) | 0.529 | Ref | 0.66 (0.28, 1.55) | 1.01 (0.43, 2.34) | 0.988 |
| Model 2 | 1.14 (0.81, 1.59) | 0.456 | Ref | 0.70 (0.30, 1.65) | 1.08 (0.46, 2.53) | 0.864 |
| *PFPeS* |  |  |  |  |  |  |
| Crude | 1.08 (0.71, 1.63) | 0.720 | Ref | 1.45 (0.65, 3.23) | 1.45 (0.62, 3.40) | 0.373 |
| Model 1 | 1.09 (0.71, 1.67) | 0.689 | Ref | 1.43 (0.64, 3.21) | 1.50 (0.63, 3.59) | 0.341 |
| Model 2 | 1.10 (0.72, 1.68) | 0.666 | Ref | 1.43 (0.63, 3.21) | 1.57 (0.66, 3.78) | 0.296 |
| *6m-PFOS* |  |  |  |  |  |  |
| Crude | 1.06 (0.74, 1.51) | 0.763 | Ref | 0.82 (0.36, 1.91) | 1.25 (0.55, 2.82) | 0.596 |
| Model 1 | 1.05 (0.73, 1.51) | 0.812 | Ref | 0.77 (0.32, 1.84) | 1.23 (0.54, 2.83) | 0.605 |
| Model 2 | 1.06 (0.73, 1.52) | 0.770 | Ref | 0.79 (0.33, 1.89) | 1.28 (0.56, 2.95) | 0.545 |
| *3,4,5m-PFOS* |  |  |  |  |  |  |
| Crude | 0.92 (0.67, 1.28) | 0.625 | Ref | 0.78 (0.34, 1.78) | 1.08 (0.47, 2.48) | 0.868 |
| Model 1 | 0.92 (0.65, 1.28) | 0.614 | Ref | 0.72 (0.31, 1.70) | 1.10 (0.47, 2.57) | 0.833 |
| Model 2 | 0.92 (0.66, 1.29) | 0.622 | Ref | 0.72 (0.31, 1.71) | 1.10 (0.47, 2.58) | 0.819 |
| *1m-PFOS* |  |  |  |  |  |  |
| Crude | 0.83 (0.61, 1.14) | 0.252 | Ref | 0.86 (0.38, 1.95) | 0.77 (0.33, 1.79) | 0.547 |
| Model 1 | 0.83 (0.60, 1.15) | 0.258 | Ref | 0.83 (0.36, 1.92) | 0.80 (0.34, 1.86) | 0.596 |
| Model 2 | 0.84 (0.60, 1.16) | 0.293 | Ref | 0.86 (0.37, 1.99) | 0.83 (0.35, 1.95) | 0.660 |
| *Br-PFHxS* |  |  |  |  |  |  |
| Crude | 0.82 (0.61, 1.09) | 0.168 | Ref | 0.71 (0.31, 1.62) | 0.68 (0.29, 1.56) | 0.357 |
| Model 1 | 0.82 (0.61, 1.11) | 0.196 | Ref | 0.70 (0.30, 1.63) | 0.71 (0.30, 1.67) | 0.423 |
| Model 2 | 0.82 (0.61, 1.11) | 0.200 | Ref | 0.71 (0.30, 1.66) | 0.72 (0.30, 1.70) | 0.448 |
| ***PFCA*** |  |  |  |  |  |  |
| *PFOA* |  |  |  |  |  |  |
| Crude | 0.84 (0.62, 1.14) | 0.265 | Ref | 0.50 (0.21, 1.16) | 0.72 (0.32, 1.63) | 0.424 |
| Model 1 | 0.81 (0.59, 1.11) | 0.186 | Ref | 0.51 (0.22, 1.19) | 0.68 (0.30, 1.56) | 0.344 |
| Model 2 | 0.82 (0.60, 1.12) | 0.210 | Ref | 0.52 (0.22, 1.23) | 0.70 (0.30, 1.62) | 0.382 |
| *PFNA* |  |  |  |  |  |  |
| Crude | 1.00 (0.69, 1.44) | 0.997 | Ref | 1.28 (0.55, 2.98) | 1.05 (0.47, 2.36) | 0.900 |
| Model 1 | 0.96 (0.66, 1.40) | 0.850 | Ref | 1.25 (0.53, 2.97) | 0.97 (0.42, 2.25) | 0.940 |
| Model 2 | 0.96 (0.66, 1.40) | 0.841 | Ref | 1.31 (0.55, 3.11) | 0.97 (0.42, 2.25) | 0.945 |
| *PFDA* |  |  |  |  |  |  |
| Crude | 1.07 (0.78, 1.46) | 0.670 | Ref | 1.85 (0.80, 4.28) | 1.58 (0.69, 3.61) | 0.280 |
| Model 1 | 1.03 (0.75, 1.43) | 0.853 | Ref | 1.75 (0.73, 4.20) | 1.49 (0.62, 3.57) | 0.391 |
| Model 2 | 1.04 (0.75, 1.45) | 0.807 | Ref | 1.81 (0.75, 4.37) | 1.55 (0.65, 3.72) | 0.347 |
| *PFHxA* |  |  |  |  |  |  |
| Crude | 1.07 (0.79, 1.44) | 0.683 | Ref | 0.73 (0.32, 1.66) | 1.39 (0.61, 3.16) | 0.462 |
| Model 1 | 1.08 (0.80, 1.46) | 0.624 | Ref | 0.78 (0.33, 1.84) | 1.48 (0.64, 3.44) | 0.367 |
| Model 2 | 1.06 (0.78, 1.45) | 0.693 | Ref | 0.79 (0.33, 1.86) | 1.45 (0.62, 3.38) | 0.401 |
| *PFBA* |  |  |  |  |  |  |
| Crude | 0.98 (0.80, 1.20) | 0.846 | Ref | 1.16 (0.52, 2.60) | 1.48 (0.63, 3.47) | 0.367 |
| Model 1 | 0.98 (0.80, 1.20) | 0.858 | Ref | 1.20 (0.53, 2.71) | 1.52 (0.65, 3.59) | 0.339 |
| Model 2 | 0.99 (0.81, 1.21) | 0.893 | Ref | 1.26 (0.55, 2.89) | 1.50 (0.63, 3.54) | 0.354 |
| *PFPeA* |  |  |  |  |  |  |
| Crude | 0.97 (0.70, 1.34) | 0.857 | Ref | 0.57 (0.25, 1.32) | 1.07 (0.46, 2.46) | 0.899 |
| Model 1 | 0.97 (0.70, 1.35) | 0.873 | Ref | 0.57 (0.25, 1.31) | 1.07 (0.46, 2.47) | 0.909 |
| Model 2 | 0.96 (0.69, 1.34) | 0.814 | Ref | 0.57 (0.25, 1.31) | 1.01 (0.43, 2.37) | 0.992 |
| *PFTrDA* |  |  |  |  |  |  |
| Crude | 1.15 (0.84, 1.57) | 0.372 | Ref | 1.45 (0.64, 3.28) | 1.18 (0.52, 2.71) | 0.671 |
| Model 1 | 1.13 (0.82, 1.55) | 0.452 | Ref | 1.44 (0.63, 3.31) | 1.12 (0.48, 2.59) | 0.772 |
| Model 2 | 1.14 (0.83, 1.58) | 0.414 | Ref | 1.41 (0.61, 3.26) | 1.12 (0.48, 2.61) | 0.767 |
| *PFTeDA* |  |  |  |  |  |  |
| Crude | 0.97 (0.68, 1.38) | 0.850 | Ref | 0.62 (0.26, 1.44) | 1.10 (0.49, 2.49) | 0.804 |
| Model 1 | 0.99 (0.69, 1.41) | 0.938 | Ref | 0.61 (0.26, 1.42) | 1.11 (0.49, 2.51) | 0.789 |
| Model 2 | 0.97 (0.67, 1.39) | 0.849 | Ref | 0.61 (0.26, 1.42) | 1.09 (0.48, 2.48) | 0.810 |
| *PFUnDA* |  |  |  |  |  |  |
| Crude | 1.13 (0.83, 1.53) | 0.452 | Ref | 1.02 (0.45, 2.32) | 1.14 (0.50, 2.62) | 0.762 |
| Model 1 | 1.09 (0.79, 1.50) | 0.595 | Ref | 0.98 (0.41, 2.33) | 1.04 (0.44, 2.46) | 0.929 |
| Model 2 | 1.11 (0.80, 1.55) | 0.524 | Ref | 0.97 (0.41, 2.30) | 1.07 (0.45, 2.56) | 0.891 |
| ***Short-Chain and Emerging Replacement PFAS*** | | |  |  |  |  |
| *4:2 FTS* |  |  |  |  |  |  |
| Crude | 0.91 (0.60, 1.36) | 0.637 | Ref | 0.95 (0.42, 2.16) | 0.76 (0.33, 1.77) | 0.530 |
| Model 1 | 0.91 (0.61, 1.38) | 0.669 | Ref | 0.93 (0.40, 2.12) | 0.77 (0.33, 1.78) | 0.538 |
| Model 2 | 0.92 (0.61, 1.39) | 0.694 | Ref | 0.91 (0.40, 2.09) | 0.78 (0.34, 1.81) | 0.565 |
| *6:2 Cl-PFESA* |  |  |  |  |  |  |
| Crude | 0.98 (0.74, 1.29) | 0.889 | Ref | 1.13 (0.50, 2.58) | 1.20 (0.52, 2.77) | 0.668 |
| Model 1 | 0.97 (0.73, 1.29) | 0.828 | Ref | 1.12 (0.49, 2.57) | 1.20 (0.52, 2.78) | 0.667 |
| Model 2 | 0.98 (0.74, 1.31) | 0.908 | Ref | 1.18 (0.51, 2.72) | 1.23 (0.53, 2.85) | 0.624 |
| *8:2 Cl-PFESA* |  |  |  |  |  |  |
| Crude | 0.97 (0.74, 1.26) | 0.810 | Ref | 0.77 (0.33, 1.77) | 0.60 (0.26, 1.37) | 0.223 |
| Model 1 | 0.95 (0.73, 1.25) | 0.734 | Ref | 0.75 (0.32, 1.74) | 0.59 (0.25, 1.36) | 0.215 |
| Model 2 | 0.95 (0.72, 1.24) | 0.685 | Ref | 0.76 (0.32, 1.81) | 0.57 (0.24, 1.32) | 0.185 |
| *HFPO-DA* |  |  |  |  |  |  |
| Crude | 2.68 (1.41, 5.08) ^**^ | 0.003 | Ref | 2.02 (0.88, 4.66)^*^ | 2.27 (0.97, 5.34)^*^ | 0.059 |
| Model 1 | 2.78 (1.41, 5.50) ^**^ | 0.003 | Ref | 1.96 (0.84, 4.57) | 2.21 (0.89, 5.54)^*^ | 0.079 |
| Model 2 | 2.76 (1.39, 5.46)^**^ | 0.004 | Ref | 1.87 (0.80, 4.41) | 2.14 (0.85, 5.38) | 0.097 |

^*^Associations were estimated using ordinal logistic regression models for each PFAS. PFAS concentrations were natural logarithm-transformed before analysis. Odds ratios and 95% confidence intervals represent the association with more advanced TNM stage per one-unit increase in ln-transformed PFAS concentration. The crude model was unadjusted; Model 1 was adjusted for sex, age, and BMI; and Model 2 was further adjusted for smoking status and drinking status. ^*^ P < 0.10; ^**^ P < 0.05.

**Table S9. Posterior inclusion probabilities of all 23 PFAS in the sensitivity BKMR analysis of TNM stage in colorectal cancer.**

|  | PIP | |
| --- | --- | --- |
|  | Model 1 | Model 2 |
| ***PFSA*** |  |  |
| FOSA | 0.075 | 0.099 |
| PFBS | 0.012 | 0.012 |
| PFHpS | 0.032 | 0.019 |
| n- PFHxS | 0.044 | 0.021 |
| n-PFOS | 0.041 | 0.014 |
| PFPeS | 0.021 | 0.013 |
| 6m-PFOS | 0.029 | 0.054 |
| 3,4,5m-PF0S | 0.044 | 0.003 |
| 1m-PFOS | 0.086 | 0.048 |
| Br- PFHxS | 0.086 | 0.069 |
| ***PFCA*** |  |  |
| PFOA | 0.075 | 0.067 |
| PFNA | 0.014 | 0.012 |
| PFDA | 0.021 | 0.010 |
| PFHxA | 0.028 | 0.003 |
| PFBA | 0.010 | 0.017 |
| PFPeA | 0.035 | 0.053 |
| PFTrDA | 0.063 | 0.027 |
| PFTeDA | 0.048 | 0.001 |
| PFUnDA | 0.009 | 0.084 |
| ***Short-Chain and Emerging Replacement PFAS*** | |  |
| 4:2 FTS | 0.004 | 0.033 |
| 6:2 Cl-PFESA | 0.069 | 0.116 |
| 8:2 Cl-PFESA | 0.070 | 0.086 |
| HFPO-DA | 0.256 | 0.241 |

^*^All 23 PFAS with detection frequencies greater than 70% were included simultaneously in the BKMR model. Model 1 was adjusted for sex, age, and BMI; Model 2 was further adjusted for smoking status and drinking status. Higher posterior inclusion probabilities indicate greater relative contributions to the overall mixture association.

**Table S10. Sensitivity analysis of the associations between the five selected PFAS and TNM stage in colorectal cancer after excluding extreme observations (N=88).**

| PFAS | Continuous PFAS levels | |  | Categorical PFAS levels | | | |
| --- | --- | --- | --- | --- | --- | --- | --- |
|  | OR (95% CI) | P |  | Q1 | Q2 | Q3 | P_-trend_ |
| ***PFPeS*** |  |  |  |  |  |  |  |
| Crude | 0.99 (0.55, 1.78) | 0.984 |  | Ref | 1.27 (0.55, 2.91) | 1.32 (0.55, 3.13) | 0.525 |
| Model 1 | 1.00 (0.55, 1.82) | 0.995 |  | Ref | 1.25 (0.54, 2.89) | 1.37 (0.57, 3.30) | 0.483 |
| Model 2 | 0.99 (0.54, 1.79) | 0.963 |  | Ref | 1.24 (0.54, 2.88) | 1.36 (0.57, 3.30) | 0.483 |
| ***PFTrDA*** |  |  |  |  |  |  |  |
| Crude | 1.21 (0.85, 1.72) | 0.298 |  | Ref | 1.12 (0.48, 2.59) | 1.21 (0.52, 2.80) | 0.66 |
| Model 1 | 1.18 (0.82, 1.70) | 0.377 |  | Ref | 1.10 (0.46, 2.59) | 1.13 (0.48, 2.68) | 0.777 |
| Model 2 | 1.20 (0.83, 1.74) | 0.329 |  | Ref | 1.08 (0.46, 2.57) | 1.16 (0.48, 2.77) | 0.743 |
| ***PFTeDA*** |  |  |  |  |  |  |  |
| Crude | 0.86 (0.57, 1.31) | 0.491 |  | Ref | 0.68 (0.29, 1.59) | 0.96 (0.42, 2.21) | 0.937 |
| Model 1 | 0.87 (0.57, 1.32) | 0.513 |  | Ref | 0.66 (0.28, 1.57) | 0.97 (0.42, 2.22) | 0.944 |
| Model 2 | 0.85 (0.56, 1.30) | 0.461 |  | Ref | 0.66 (0.28, 1.57) | 0.96 (0.42, 2.20) | 0.934 |
| ***8:2 Cl-PFESA*** |  |  |  |  |  |  |  |
| Crude | 0.96 (0.67, 1.39) | 0.839 |  | Ref | 0.74 (0.30, 1.79) | 0.83 (0.35, 1.97) | 0.679 |
| Model 1 | 0.93 (0.64, 1.34) | 0.69 |  | Ref | 0.68 (0.27, 1.67) | 0.79 (0.33, 1.90) | 0.611 |
| Model 2 | 0.94 (0.65, 1.37) | 0.762 |  | Ref | 0.68 (0.27, 1.68) | 0.81 (0.34, 1.95) | 0.649 |
| ***HFPO-DA*** |  |  |  |  |  |  |  |
| Crude | 2.54 (1.32, 4.87) | 0.005 |  | Ref | 2.04 (0.88, 4.73)^*^ | 2.14 (0.92, 5.00)^*^ | 0.080 |
| Model 1 | 2.65 (1.33, 5.28)^**^ | 0.005 |  | Ref | 2.01 (0.86, 4.70) | 2.12 (0.85, 5.31) | 0.093 |
| Model 2 | 2.62 (1.31, 5.24)^**^ | 0.006 |  | Ref | 1.89 (0.80, 4.51) | 2.07 (0.82, 5.18) | 0.110 |

^*^Extreme observations were defined as PFAS concentrations exceeding the third quartile plus three times the interquartile range on the original, untransformed scale. For each PFAS, observations exceeding the compound-specific threshold were excluded before analysis. Odds ratios and 95% confidence intervals were estimated using ordinal logistic regression models. PFAS concentrations were natural logarithm-transformed before analysis. The crude model was unadjusted; Model 1 was adjusted for sex, age, and BMI; and Model 2 was further adjusted for smoking status and drinking status. ^*^ P < 0.10; ^**^ P < 0.05.

**Table S11. Posterior inclusion probabilities of the five PFAS in BKMR models after excluding extreme observations (N=88).**

|  | PIP | |
| --- | --- | --- |
|  | Model 1 | Model 2 |
| PFPeS | 0.328 | 0.320 |
| PFTrDA | 0.190 | 0.190 |
| PFTeDA | 0.118 | 0.129 |
| HFPO-DA | 0.345 | 0.359 |
| 8:2 Cl-PFESA | 0.277 | 0.275 |

^*^Participants with a concentration exceeding the third quartile plus three times the interquartile range for any of the five PFAS on the original, untransformed scale were excluded before analysis. Model 1 was adjusted for sex, age, and BMI; Model 2 was further adjusted for smoking status and drinking status. Higher posterior inclusion probabilities indicate greater relative contributions of individual PFAS to the overall mixture association.

**Figure S1. Spearman correlation of plasma PFAS.**

**Figure S2. Association between ln-transformed PFAS levels and Tumor TNM Stage of Colorectal Cancer analyzed using Restricted Cubic Spline Models.**

^*^ Solid lines represent the estimated exposure–response associations, and shaded areas indicate 95% confidence intervals. The median concentration of each PFAS was used as the reference. P values for overall and nonlinear associations are shown in each panel. Models were adjusted for sex, age, and BMI.

**Figure S3. Subgroup analysis: Joint effect estimates with 95% CI for the overall mixture in relation to Tumor TNM Stage of Colorectal Cancer in BKMR models stratified by sex.**

^*^(A) Estimated overall association of the five-PFAS mixture with TNM stage when all PFAS were jointly fixed at selected exposure quantiles, relative to their median concentrations. Points represent posterior estimates, and error bars represent 95% credible intervals. (B) Posterior inclusion probabilities of the five PFAS in the BKMR model. (C) Estimated associations of individual PFAS with TNM stage when the remaining mixture components were fixed at the 25th, 50th, or 75th percentiles. Results are presented separately for male and female patients. Models were adjusted for age and BMI.

**Figure S4. Subgroup analysis: Joint effect estimates with 95% CI for the overall mixture in relation to Tumor TNM Stage of Colorectal Cancer in BKMR models stratified by age.**

^*^(A) Estimated overall mixture association with TNM stage when all five PFAS were jointly fixed at selected exposure quantiles, relative to their median concentrations. Points represent posterior estimates, and error bars represent 95% credible intervals. (B) Posterior inclusion probabilities of the five PFAS in the BKMR model. (C) Estimated associations of individual PFAS with TNM stage when the remaining mixture components were fixed at the 25th, 50th, or 75th percentiles. Results are presented separately for patients aged ≥65 years and <65 years. Models were adjusted for sex and BMI.

**Figure S5. Sensitivity analysis of the association between the 23-PFAS mixture and TNM stage in colorectal cancer using BKMR.**

*(A) Estimated overall mixture association with TNM stage when all 23 PFAS were jointly fixed at selected exposure quantiles, relative to their median concentrations. Points represent posterior estimates, and error bars represent 95% credible intervals. (B) Posterior inclusion probabilities of the 23 PFAS in the BKMR model. (C) Estimated associations of individual PFAS with TNM stage when the remaining mixture components were fixed at the 25th, 50th, or 75th percentiles. The model was adjusted for sex, age, BMI, smoking status, and drinking status.

**Figure S6. Sensitivity analysis of the association between the five-PFAS mixture and TNM stage in colorectal cancer after excluding extreme observations using BKMR (N=88).**

^*^(A) Estimated overall association of the five-PFAS mixture with TNM stage when all PFAS were jointly fixed at selected exposure quantiles, relative to their median concentrations. Points represent posterior estimates, and error bars represent 95% credible intervals. (B) Posterior inclusion probabilities of the five PFAS. (C) Estimated associations of individual PFAS with TNM stage when the remaining mixture components were fixed at the 25th, 50th, or 75th percentiles. Extreme observations were defined as concentrations exceeding the third quartile plus three times the interquartile range on the original, untransformed scale. Participants with an extreme concentration in any of the five PFAS were excluded. Model 1 was adjusted for sex, age, and BMI; Model 2 was further adjusted for smoking status and drinking status.
